# The Relationship between Diabetic Neuropathy and Sleep Apnea Syndrome: A Meta-Analysis

**DOI:** 10.1155/2013/150371

**Published:** 2013-12-07

**Authors:** Kazuya Fujihara, Satoru Kodama, Chika Horikawa, Sakiko Yoshizawa, Ayumi Sugawara, Reiko Hirasawa, Hitoshi Shimano, Yoko Yachi, Akiko Suzuki, Osamu Hanyu, Hirohito Sone

**Affiliations:** ^1^Department of Internal Medicine, Faculty of Medicine, Tsukuba University, Japan; ^2^Department of Internal Medicine, Faculty of Medicine, Niigata University, 1-754 Asahimachi, Niigata, Niigata 951-8510, Japan

## Abstract

*Aims*. High prevalence of sleep apnea syndrome (SAS) has been reported in patients with diabetes. However, whether diabetic neuropathy (DN) contributes to this high prevalence is controversial. Our aim of this study is to compare the prevalence of SAS between patients with and without DN. *Methods*. Systematic literature searches were conducted for cross-sectional studies that reported the number of patients with DN and SAS using MEDLINE (from 1966 to Nov 5, 2012) and EMBASE (from 1974 to Nov 5, 2012). Odds ratios (ORs) of SAS related to DN were pooled with the Mantel-Haenszel method. *Results*. Data were obtained from 5 eligible studies (including 6 data sets, 880 participants, and 429 cases). Overall, the pooled OR of SAS in patients with DN compared with that in non-DN patients was significant (OR (95% CI), −1.95 (1.03–3.70)). The pooled OR of SAS was 1.90 (0.97–3.71) in patients with type 2 diabetes. Excluding data on patients with type 1 diabetes, a higher OR was observed in younger patients (mean age <60 years) than in those ≥60 years among whom the OR remained significant (3.82; 95% CI, 2.24–6.51 and 1.17; 95% CI, 0.81–1.68). *Conclusions*. Current meta-analysis suggested the association of some elements of neuropathy with SAS in type 2 diabetes. Further investigations are needed to clarify whether the association is also true for patients with type 1 diabetes.

## 1. Introduction

Sleep apnea syndrome (SAS) is characterized by nocturnal sleep restriction, sleep fragmentation, and intermittent hypoxia, resulting in poor sleep quality and daytime sleepiness [1, 2]. The prevalence of SAS, in particular obstructive sleep apnea, is dramatically increasing with the increased prevalence of obesity, which is the main cause of the upper airway obstruction typically observed as snoring while sleeping [3]. SAS not only causes a lower quality of life due to sleepiness but also has clinical consequences that include hypertension, diabetes, cardiovascular disease, and sudden death [1, 2, 4].

A recent meta-analysis indicated that obstructive sleep apnea is associated with an increased risk of future type 2 diabetes, [5] clearly suggesting that individuals with diabetes had a higher prevalence of SAS compared to those without diabetes. The higher prevalence of SAS is partially explained by the higher prevalence of obesity among individuals with diabetes compared with those without diabetes [6, 7]. Diabetic neuropathy (DN) [8, 9] has been suggested as another explanation for the presence of SAS because it is diabetes-specific [10]. However, epidemiological findings regarding the association between DN with SAS are inconsistent [11]. Therefore, our aim of this meta-analysis is to compare the prevalence of SAS between patients with and without DN.

## 2. Materials and Methods

### 2.1. Search Strategy

Electronic literature searches (MEDLINE, from January 1966 to November 2012 and EMBASE, from January 1974 to November 2012) were conducted for studies investigating the relationships between DN and SAS. Study keywords were related to diabetes, neuropathy, and sleep apnea, which were combined using the Boolean logical operator “AND” (see Supplementary Material available online at http://dx.doi.org/10.1155/2013/150371). We added a manual search using reference lists of the relevant articles. This process was repeated until no additional articles could be identified. No language restriction was imposed.

For inclusion in the meta-analysis, a study had to fulfill the following criteria: (1) all patients had diabetes; (2) cross-sectional design was used; and (3) data on the number of cases and noncases of SAS according to the prevalence of DN were provided. However, we limited the meta-analysis to studies in which the presence of SAS was determined by instruments such as the apnea hypoxia index (AHI) or respiratory disturbance index (RDI) or pulse oximetry that calculated the oxygen desaturation index (ODI: number of desaturation events ≥4%/h). Therefore, we excluded one study [12] because the presence of SAS was judged by the existence of breathing pauses while sleeping and another study [13] because the criteria of SAS were not clarified. We also excluded studies that did not specify the type of diabetes [12, 14–17] because the characteristics of neuropathy, such as severity, resulting from type 1 diabetes and type 2 diabetes are different [18].

### 2.2. Data Extraction

Two of the investigators (Kazuya Fujihara and Satoru Kodama) independently identified eligible studies and reviewed all relevant articles, including extraction of all relevant data and assessment of study quality. Discrepancies were resolved by the third investigator (Hirohito Sone). We extracted the following data from each publication: the first author's name, year of publication, geographic region, type of diabetes, participants' characteristics (i.e., age (mean), body mass index (mean), and duration of diabetes (mean)), proportion of men, definitions of SAS, number of SAS categories, type of DN (autonomic or peripheral), definitions of DN, number of participants and cases, and adjustment for age and sex or for obesity-related variables. Quality assessment was conducted by modifying the Newcastle-Ottawa Scale (NOS) for case-control studies so that it would be applicable to this meta-analysis [19]. Supplementary Material 2 shows the questions used in this assessment. Each “Yes” answer was awarded one point, with 8 as the highest possible score. Study quality was judged by the total of awarded points as follows: low (<4 points) and high (≥4 points).

### 2.3. Data Synthesis

Odds ratios (ORs) for DN related to SAS were pooled with the Mantel-Haenszel method using DerSimonian and Laird's random-effects model [20]. This model considers between-study heterogeneity assessed by *I*-squared [21]. Primarily, in pooling the relationship between DN and SAS, we gave priority to SAS diagnosed using AHI if data on both the prevalence of abnormal AHI and ODI were given [22]. In addition, priority was given to data on mild SAS cases if multiple data were provided according to the severity of SAS using AHI or ODI [22–24].

Analyses were stratified by the following prespecified confounders that potentially influenced the study results: mean age (≥60 or <60 years); mean BMI (≥30 kg/m^2^ or <30 kg/m^2^); country (Asian or non-Asian); type of DN (autonomic, peripheral, or unknown); and study quality (high or low). To explore the effect of study characteristics on the risk of SAS, meta-regression analysis was conducted where each confounder that we described above was entered as an explanatory variable and log OR as a dependent variable. Publication bias was statistically assessed using Egger's regression test [25]. A two-sided *P* value less than 0.05 was statistically significant. All analyses were conducted with STATA software version 11 (STATA Corporation, College Station, TX, USA).

## 3. Results

### 3.1. Literature Research

Supplementary Material 3 shows details of the literature searches. First, 270 citations were identified. Of these, 237 articles were excluded according to their titles and abstracts. Forty articles, including 7 articles obtained from the manual search, were included for a more detailed review. After this review, 35 were excluded for the reasons shown in Supplementary Material 3. Finally, 5 eligible studies were included in this meta-analysis.

### 3.2. Study Characteristics

Characteristics of the 5 selected studies comprising 6 data sets, 880 participants, 442 cases of DN, and 429 cases of SAS are shown in Table 1. All 5 studies included both males and females [22–24, 26, 27]. Mean age of participants was under 60 years in 3 studies [22, 24, 26] and was 60 years or over in 2 studies [23, 27]. Two studies [24, 27] were of the Asian region and 3 of non-Asian regions [22, 23, 26]. Mean duration was over 10 years in all 5 studies [22–24, 26, 27]. Three studies [22, 24, 26] used the AHI to diagnose SAS, whereas 1 study [27] used the ODI. One study [23] used the RDI. Among the 5 included studies, 1 [22] and 3 [23, 26, 27] investigated participants with autonomic neuropathy and peripheral neuropathy, respectively. According to the NOS scale, 3 studies were judged to be of high quality [22, 26, 27] and the other 2 were judged to be of low quality [23, 24] (Supplementary Material 2). One study [22] separately allowed estimation of the OR for SAS based on type 1 and type 2 diabetes. These ORs were separately pooled after the overall analysis. We first calculated the OR for both type 1 diabetes and type 2 diabetes, then calculated the OR for only type 2 diabetes since we only obtained data from one study for type 1 diabetes.

### 3.3. Overall Estimate of Prevalence of Sleep Apnea Syndrome Associated with Diabetic Neuropathy

**Figure 1:**
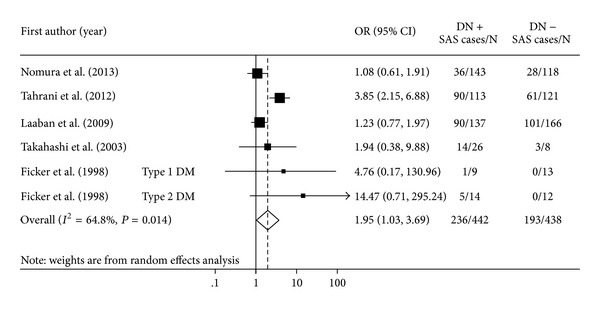
Forest plot showing the odds ratios (ORs) with 95% confidence interval (95% CI) of sleep apnea syndrome (SAS) for participants with diabetic neuropathy (DN) compared to participants without DN. Pooled OR is indicated by a diamond. The 95% CI of each OR is indicated by a vertical line. Size of squares reflects the statistical weight of each study.

**Figure 2:**
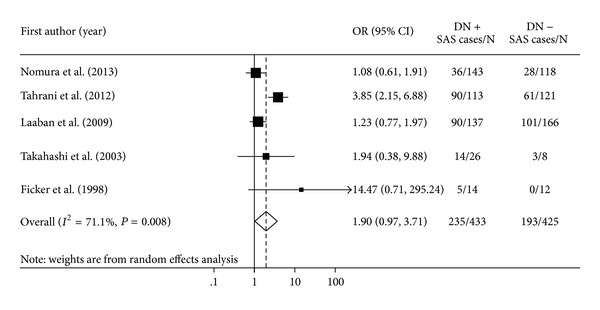
Forest plot showing the odds ratios (ORs) with 95% confidence interval (95% CI) of sleep apnea syndrome (SAS) for participants with diabetic neuropathy (DN) compared to participants without DN. Pooled OR is indicated by a diamond. The 95% CI of each OR is indicated by a vertical line. Size of squares reflects the statistical weight of each study.

Figure 1 shows the pooled estimates for SAS in persons with DN. The overall pooled OR of SAS for DN participants compared with that for the non-DN participants was 1.95 (95% CI, 1.03–3.69; *P* < 0.041). However, between-study heterogeneity in the strength of the association was highly significant (*I* ^2^ = 64.8%; *P* = 0.014). After excluding one data set on patients with type 1 diabetes expressing 4.76 of OR for SAS, the overall pooled OR of SAS for DN participants compared with that for the non-DN participants was 1.90 (95% CI, 0.97–3.71 *P* = 0.060) in those with type 2 diabetes (Figure 2).

### 3.4. Sensitivity Analysis

Table 2 and Supplementary Material 4 show the results of stratified and meta-regression analyses across a number of key study characteristics to explore the origin of the heterogeneity and the influence of those characteristics on study results. A statistically stronger association between DN and SAS prevalence was remarkable in the younger participants (mean age < 60 years) in comparison with those ≥60 years. The difference was statistically significant (3.82; 95% CI, 2.24–6.51 and 1.17; 95% CI, 0.81–1.68; *P* = 0.04) (Supplementary Material 4 and Table 2).

### 3.5. Publication Bias

Egger's test revealed that there was no publication bias in both overall analysis and studies of only type 2 diabetes (*P* = 0.42 and *P* = 0.50, resp.).

## 4. Discussion

The current meta-analysis indicated that DN patients had approximately 2-fold higher prevalence of SAS than diabetic patients without neuropathy. Every 10 years of aging has been associated with a 1.24-fold increased risk of SAS in the general population [28]. If these data were applied to the current results, DN individuals would be estimated to develop SAS more than 30 years earlier than diabetic patients without neuropathy. Another main finding of the stratified analysis was that the effect of DN on the prevalence of SAS was remarkable in studies targeting relatively young diabetic patients (OR = 3.8) compared with those targeting relatively elderly patients (OR = 1.2). This result could be interpreted to mean that the association between DN and SAS is prominent in a young diabetic population among which the effect of aging is relatively weak, whereas an association between DN and SAS might be masked by the effect of aging in the more elderly patients. Consequently, it is suggested that DN was significantly associated with high prevalence of SAS without regard to the effect of aging.

A meta-analysis of observational studies in principle can never prove causality. However, there are possible mechanisms as to why both autonomic and peripheral DN could lead to progression of SAS. An association between impairment of autonomic dysfunction and SAS was reported. The impairment in the central generation of respiratory movements that have been seen in autonomic disorders, such as Shy-Drager syndrome, may lead to collapse of the upper airway [29]. Another mechanism is that the reduction in sympathetic afferent nerves from the lung or CO_2_-induced sympathetic activity controlling the ventilatory output may lead to an enhanced response to hypercapnia [30]. This abnormal respiratory control may account for central sleep apnea [12]. On the other hand, the association between impairment of peripheral nerve dysfunction and SAS also has been reported. The impairment of the pharyngeal sensory nerve, which fails to compensate for the compromised upper airway through protective reflexes, thereby increasing upper airway dilating muscle activity, finally leads to pharyngeal collapse and obstructive sleep apnea [31]. This impairment is known to be observed in a generalized neuropathy such as Charcot-Marie-Tooth [32]. Interestingly, deterioration of the reflexes protecting the upper airway is known to correlate with aging [33]. Another possible mechanism is that painful peripheral neuropathy might disturb sleep. However, it is obvious that the detailed mechanism elucidating the relationship between DN and SAS should be studied in the future.

*Study Limitations.* Several limitations need be addressed regarding this meta-analysis. First, this meta-analysis could not access individual data in each included study because it was study-based but not individual-based. Therefore, it is impossible to perfectly control for confounders linking DN and SAS. In addition, unfortunately, adjustment for possible residual confounders (e.g., cognitive heart failure, smoking, glycemic control, and dyslipidemia) [1, 34–36] in each included study was generally poor. For example, higher prevalence of chronic heart failure or poor glycemic control was observed in patients with DN than that in patients without DN [1, 34–36]. The failure of adjustment for these confounders might have been responsible for the apparent association between DN and SAS. Second, meta-analyses of cross-sectional studies do not have the ability to distinguish an exposure from an outcome. We discussed the possibility that DN was associated with incident SAS. However, a reverse association could not be ruled out. A recent meta-analysis indicated that obstructive sleep apnea is associated with an increased risk of type 2 diabetes [5]. Therefore, it is possible that patients having SAS had developed diabetes and, at a later time, developed DN due to poor diabetes care. Third, few of the studies randomly selected their participants. It might not be sufficient to ascertain external validity since subjects in these studies might not be representative of average populations of diabetic patients.

In conclusion, the results of the current meta-analysis suggested an association of elements of neuropathy with SAS in type 2 diabetes. Further investigations are needed to clarify whether the association is true in patients with type 1 diabetes.

## Supplementary Material

Supplemental Material 1. Study keywords in this meta-analysis. Study keywords were related to diabetes, neuropathy, and sleep apnea, which were combined using the
Supplemental Material 2. Results of quality assessment of the included studies using the modified Newcastle-Ottawa Scale.
Supplemental Material 3. Flowchart of Meta-analysis.
Supplemental Material 4. Forest plot showing the odds ratios (ORs) with 95% confidence intervals (95% CI) of sleep apnea syndrome for participants with type 2 diabetes who have diabetic neuropathy (DN) compared to participants without DN. Pooled OR is indicated by a diamond. A, B, C, D, and E show the results of each forest plot with regard to age, BMI, country, type of DN, and study quality category, respectively.

## Figures and Tables

**Table:** (a)

Author	Country	Type of diabetes	Age (yrs), mean	BMI (kg/m^2^), mean	Duration of diabetes (yrs)	% Men	No. of subject					SA definition	Risk group	Referent group
							Total	DN (+)	DN (+)	DN (−)	DN (−)			
								SA (+)	SA (−)	SA (+)	SA (−)			
Nomura et al. (2013) [27]	Japan	T2DM	62.8	24.9	11.6	62	261	36	107	28	90	ODI 4% ≥ 5	ODI 4% ≥ 5	ODI 4% < 5
Tahrani et al. (2012) [26]	UK	T2DM	58.0	32.9	10.3	58	234	90	23	61	60	AHI ≥ 5	AHI ≥ 5	AHI < 5
Laaban et al. (2009) [23]	France	T2DM	61.0	31.1	14.4	53	303	90	47	101	65	RDI ≥ 5	RDI ≥ 5	RDI < 5
Takahashi et al. (2003) [24]	Japan	T2DM	58.0	24.4	ND	59	34	14	12	3	5	AHI ≥ 5	AHI ≥ 5	AHI < 5
Ficker et al. (1998) [22]	Germany	T1DM 22	51.3	25.1	17.5	48	22	1	8	0	13	AHI ≥ 10	AHI ≥ 10	AHI < 10
		T2DM 26					26	5	9	0	12			

**Table:** (b)

Author	Neuropathy definition			Matched age and sex	Matched obesity
	Autonomic	Peripheral	Evaluation of diabetic neuropathy		
Nomura et al. (2013) [27]	No	Yes	DTR, vibration, dysesthesia	Yes	Yes
Tahrani et al. (2012) [26]	No	Yes	MNSI examinations scores, MNSI questionnaire scores	Yes	Yes
Laaban et al. (2009) [23]	No	Yes	Patient's records	No	No
Takahashi et al. (2003) [24]	No	No	Not described	No	No
Ficker et al. (1998) [22]	Yes	No	CV, RMSSD, *E*-*I* difference, *E*/*I* ratio, maximum/minimum 30 : 15 ratio	No	No

Abbreviations: AHI: apnea hypoxia index; BMI: body mass index; CV: coefficient of variation; DM: diabetes mellitus; DN: diabetic neuropathy, DTR: deep tendon reflexes; MNSI: Michigan neuropathy screening instrument; ODI: oxygen desaturation index; RMSSD: root mean square of successive difference; RDI: respiratory disturbance index; SA: sleep apnea; T1DM: type 1 diabetes mellitus; T2DM: type 2 diabetes mellitus.

**Table 2:** Univariate meta-regression analysis of risk of SAS related to study characteristics*.

Variable	Coefficient	SE	*P* value
Mean age ≥ 60 years	−1.16	0.33	0.04
BMI ≥ 30 kg/m^2^	0.21	0.80	0.81
Asian population	−0.65	0.74	0.44
DN diagnosis**			
Autonomic and peripheral	0.13	1.12	0.92
Autonomic only	2.14	1.72	0.34
High study quality***	0.53	0.77	0.54

*Logarithm of odds ratio for SAS was a dependent variable, and each variable was entered as an explanatory variable.

**Peripheral only was referent.

***Quality score ≥ 4 was regarded as high study quality.

Abbreviation: SE: standard error.
